# NLRP3 Gene Polymorphisms in Rheumatoid Arthritis and Primary Sjogren’s Syndrome Patients

**DOI:** 10.3390/diagnostics13020206

**Published:** 2023-01-05

**Authors:** Ruei-Nian Li, Tsan-Teng Ou, Chia-Hui Lin, Yuan-Zhao Lin, Tzu-Jung Fang, Yi-Jing Chen, Chia-Chun Tseng, Wan-Yu Sung, Cheng-Chin Wu, Jeng-Hsien Yen

**Affiliations:** 1 Department of Biomedical Science and Environmental Biology, College of Life Science, Kaohsiung Medical University, Kaohsiung 807, Taiwan; 2 Division of Rheumatology, Department of Internal Medicine, Kaohsiung Medical University Hospital, Kaohsiung 807, Taiwan; 3 Division of Geriatrics and Gerontology, Department of Internal Medicine, Kaohsiung Medical University Hospital, Kaohsiung 807, Taiwan; 4 Graduate Institute of Clinical Medicine, College of Medicine, Kaohsiung Medical University, Kaohsiung 807, Taiwan; 5 Department of Microbiology, College of Medicine, Kaohsiung Medical University, Kaohsiung 807, Taiwan; 6 Institute of Medical Science and Technology, National Sun Yat-sen University, Kaohsiung 807, Taiwan

**Keywords:** NLRP3 gene polymorphisms, rheumatoid arthritis, primary Sjogren’s syndrome

## Abstract

**Aim:** The activation of NLRP3 inflammasome leads to the stimulation of cytokines and is significantly involved in the pathogenesis and progression of autoimmune diseases. The purpose of this study is to examine the associations of NLRP3 gene polymorphisms with rheumatoid arthritis (RA) and primary Sjogren’s syndrome (SS) patients. **Methods:** A total of 239 patients with RA, 285 patients with primary SS, and 170 healthy controls were enrolled. Genomic DNA was extracted from peripheral blood mononuclear cells, and gene polymorphisms were genotyped through the TaqMan assay. Antinuclear antibody (ANA), anti-Ro, and anti-CCP antibodies were detected using immunofluorescence immunoassay. **Results:** The T allele of rs4612666 CT elevated the susceptibility to RA disease. The RF titer during diagnosis of RA was significantly high in RA patients with the A allele of rs12079994 G/A polymorphism. The titer of anti-CCP during diagnosis of RA was high in the absence of the C allele of rs10754558 C/G polymorphisms in RA patients. Antinuclear antibody and anti-CCP were positively associated with the A allele of rs12079994 G/A polymorphism in primary SS. The C allele of rs4612666 C/T was negatively associated with ANA in primary SS. Conclusions: The results have shown that NLRP3 gene polymorphisms may play a role in the pathogenesis of RA and primary SS.

## 1. Introduction

The nucleotide-binding oligomerization domain (NOD), leucine-rich repeat (LRR)-containing protein receptor pyrin domain containing 3 (NLRP3), is an intracellular protein complex that detects many pathogens and different stimuli. Upon activation, NLRP3 binds to apoptosis-associated speck-like protein containing a caspase-recruitment domain (ASC). Subsequently, ASC interacts with the cysteine protease pro-caspase 1 to form a multiprotein complex, the NLRP3 inflammasome [1,2]. The oligomerization of the NLRP3 complex is critical, leading to the development of inflammatory responses. The central function of NLRP3 inflammasome is to activate caspase-1 and pro-inflammatory cytokines, especially IL-1β and IL-18 [2,3]. This results in the activation of the inflammatory process and mediates pyroptotic cell death [4]. Recently, experimental studies have revealed that NLRP3 inflammasome is significantly involved in the pathogenesis and progression of autoimmune diseases, for instance, systemic lupus erythematosus (SLE), rheumatoid arthritis (RA), systemic sclerosis (SSc), Sjogren’s syndrome (SS), and inflammatory bowel disease (IBD) [1,5,6]. NLRP3 inflammasome is found in a variety of cells, such as monocytes, macrophages, neutrophils, epithelial cells, dendritic cells, T cells, B cells, fibroblasts, and keratinocytes [7,8,9,10]. High amounts of NLRP3 mRNA and high levels of NLRP3-related proteins were detected in the monocytes, macrophages, and dendritic cells of RA patients [8,11]. Mutation of the NLRP3 gene enhanced more secretion of IL-1β, and it is correlated with the progression of RA disease severity [8]. Anti-dsDNA antibodies can upregulate NLRP3 and caspase 1 in the monocytes and immune cells of SLE patients, hence, enhancing the production of IL-1β. The pathological role of NLRP3 is not clear; however, genetic polymorphisms of NLRP3 are reported to have associations with autoimmune diseases such as Muckle–Wells syndrome, chronic infantile neurological cutaneous and articular syndrome, and familial cold auto-inflammatory syndrome [12,13,14]. NLRP3 has also been reported in complex metabolic diseases mainly produced by hematopoietic cells [15].

Cytokines exert important functions in controlling immune response and are involved in excessive inflammation. Aberrant activation of NLRP3 inflammasome is implicated in exacerbating inflammation and triggering inflammatory cytokines production. Among the cytokines, IL-1β and IL-18 are two of the most studied cytokines that are cleaved by active caspase-1 [16]. Overproduction and overactivation of cytokines are involved in the pathogenesis of autoimmune diseases [17]. The NLRP3 gene is also triggered by various stimuli signals, for example, ions efflux, reactive oxygen species production, lysosome damage, and mitochondrial dysfunction in response to cellular stress response, and NLRP3 activates pathways leading to the stimulation of a variety of cytokines and inflammatory substances [16]. Pro-inflammatory cytokines, IL-1β, IL-18, and TNF, are involved in the inflammatory process of autoimmune diseases such as RA [17]. NLRP3 inflammasome activity and downstream inflammasome-related protein molecules are upregulated in RA and SS patients [8,18]. The critical roles of the NLRP3 gene in the immunopathogenesis of autoimmune diseases are still unknown. Genetic polymorphisms of NLRP3 are related to the disease susceptibility and severity of several autoimmune diseases [13,14,19,20]. RA and primary SS are chronic autoimmune diseases, and particularly IL-1β was a key cytokine in the pathogenesis of RA and primary SS [21,22,23]. Both diseases are characterized by dysregulated inflammatory responses as a result of destruction of joints and dysfunction of exocrine glands, respectively. Therefore, in this study, we chose SNP with the reference of previous reports and carried out experiments to examine the correlations of NLRP3 gene polymorphisms in RA and primary SS patients.

## 2. Methods

### 2.1. Clinical Subjects

Two hundred and thirty-nine patients of RA, two hundred and eighty-five patients of primary SS, and one hundred and seventy healthy controls were enrolled in their recruitment from the Kaohsiung Medical University Hospital. The patients with primary SS met the 2016 American College of Rheumatology (ACR) and European League Against Rheumatism (ACR/EULAR) Classification Criteria for primary SS [24]. RA was diagnosed according to the criteria set by the ACR/EULAR [25]. All patients were informed of their consent to participate in this study. This study was approved by the Institutional Review Board of Kaohsiung Medical University Hospital (KMUH).

### 2.2. Genomic DNA Extraction

Peripheral blood mononuclear cells (PBMC) were collected with Ficoll-paque (GE Healthcare) purification, and genomic DNA was extracted from the PBMC of collected patients from KMUH. The procedure is according to the commercial protocol.

### 2.3. Genotyping and Data Analysis

Four different polymorphic sites on the region of the NLRP3 gene were genotyped. NLRP3 genotyping was performed using TaqMan SNP Genotyping Assays (Applied Biosystems, Life Technologies) by real-time polymerase chain reaction (PCR) with ABI 7500 Real-Time PCR systems. The catalog number of each polymorphic site is rs12079994; c_31451917_10, rs12137901; c_26646000c_10, rs4612666; c_26646029_10, rs10754558; c_2605202810. The assay was carried out according to the protocol provided by the manufacturer.

#### Data Analysis

The association between genotypes and diseases was analyzed with the chi-square test. The association between genotypes and clinical features was analyzed with the chi-square test and Student’s *t*-test. Fisher’s Exact Test was used for determination when there was an item less than 5 in a 2 × 2 table. The odds ratio was calculated with Haldane-Ascombe correction when there was an item 0 in a 2 × 2 table. The odds ratio (OR) and its 95% confidence interval (CI) were calculated to evaluate the risk of disease. The statistical analysis was conducted using IBM SPSS Statistics version 19.

### 2.4. Detection of Autoantibodies and Rheumatoid Factor

The titer of anti-cyclic citrullinated peptide (anti-CCP) and anti-Ro antibody was detected with the EliA CCP test and ELiA Ro test, respectively, as described by the supplier (Phadia AB, Uppsala, Sweden) and anti-nucleic acid (ANA) antibody was assayed using the ANAFAST indirect fluorescence antibody kit (DiaSorin Inc., Stillwater, CO, USA). Rheumatoid factor (RF) antibody was determined with Siemens N latexRF (Siemens Healthcare Diagnostics Product, Erlangen, Germany). The assay was carried out according to the protocol provided by the manufacturer.

## 3. Results

### 3.1. T Allele of rs4612666 C/T Elevates the Susceptibility of RA

Previous studies have demonstrated that genetic polymorphism of NLRP3 was associated with susceptibility to autoimmune diseases [13,14,26]. Therefore, in this study, we have investigated experiments to determine four different polymorphic sites on the NLRP3 gene in RA and primary SS patients. The genotypes of NLRP3 gene sequence rs12079994 G/A, rs10754558 C/G, and rs12137901 T/C were not found in relation to the susceptibility of patients with RA (Table 1). However, the odds ratio (OR) of genotype rs4612666 T/T was 1.93 (*p* = 0.024, 95%CI, 1.09–3.44), the odds ratio of genotype CT + TT was 1.61 (*p* = 0.034, 95%CI, 1.04–2.49), and the OR of T allele frequency was 1.38 (*p* = 0.023, 95%CI, 1.05–1.83). The results indicated that the T allele of rs4612666 C/T was a potential risk allele and elevated the susceptibility to RA disease. On the contrary, the genotypes of NLRP3 sequence rs12079994 G/A, rs10754558 C/G, rs4612666 C/T, and rs12137901 T/C were not found in relation to the susceptibility of patients with primary SS (Table 2).

### 3.2. C Allele of Genotype TC rs12137901 Was More Susceptible to Primary SS in DR8-Negative Individuals

Kang et al. [27] and Miyagawa et al. [28] stated that HLA-DR8 was related to pSS in Chinese and Japanese patients, respectively; therefore, we analyzed the NLRP3 genotype in the HLA-DR8 subpopulation. The OR of genotype TC + CC rs12137901 was 1.79 (*p* = 0.04, 95%CI, 1.04–3.09), and the OR of the C allele frequency was 1.58 (*p* = 0.037, 95%CI, 1.04–2.40) in HLA-DR8 negative primary SS patients (Table 3). The result indicated that the C allele of genotype TC rs12137901 was more susceptible to primary SS in HLA-DR8-negative individuals.

### 3.3. Production of Autoantibodies Were Related to RA and Primary SS Patients

**Figure 1:**
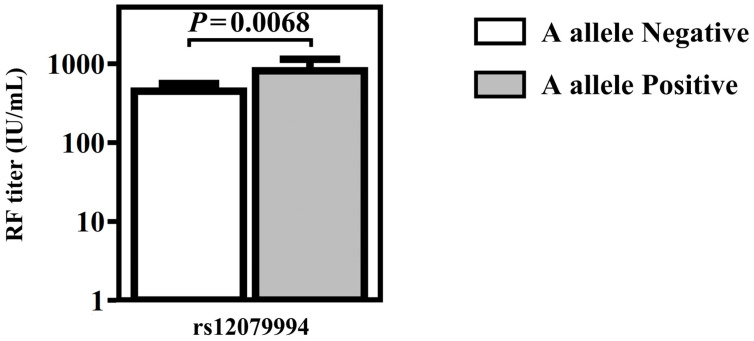
Associations of rs12079994 G/A polymorphisms with titer of rheumatoid factor.

**Figure 2:**
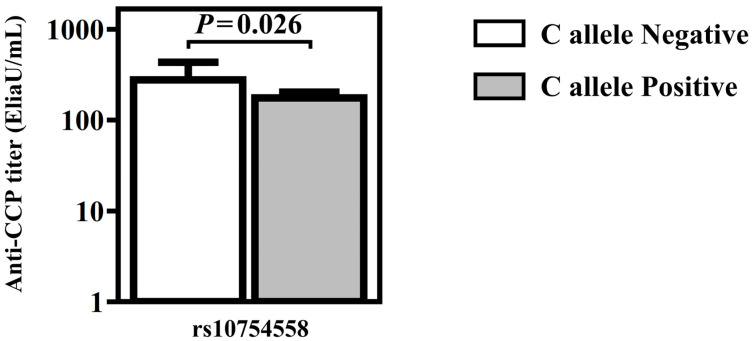
Anti-CCP antibody titer in rs10754558 C/G polymorphisms in RA patients.

The RF titer during diagnosis of RA was 813.04 ± 1239.01 IU/mL in RA patients with the A allele of rs12079994 G/A and 446.92 ± 718.03 IU/mL in RA patients without the A allele of rs12079994 G/A (Figure 1). The RF titer during diagnosis of RA was significantly high in RA patients with A allele of rs12079994 G/A polymorphism. The titer of anti-CCP during diagnosis of RA was 275.98 ± 333.76 EliaU/mL in RA patients without the C allele of rs10754558 C/G and 175.36 ± 151.72 EliaU/mL in RA patients with C allele positive of rs10754558 C/G polymorphism (Figure 2). The titer of anti-CCP during diagnosis of RA was high in the absence of the C allele of rs10754558 C/G polymorphism in RA patients. In primary SS patients, the OR of A allele positive of genotype rs12079994 G/A was 4.24 (*p* = 0.011, 95%CI, 1.29–13.99) with anti-CCP antibody (Table 4). C allele negative of genotype rs4612666 C/T was in the high odds ratio of anti-CCP antibody, but without statistical significance (Table 4). The OR of A allele positive of genotype rs12079994 G/A was 2.02 (*p* = 0.044, 95%CI, 1.01–4.03) in primary SS patients with ANA antibody (Table 5). Antinuclear antibody and anti-CCP were positively associated with the A allele of rs12079994 G/A polymorphism in primary SS. Alternatively, the OR of C allele negative of genotype rs4612666 T/C was 2.34 (*p* = 0.018, 95%CI, 1.15–4.77) in primary SS patients with ANA antibody (Table 5). The C allele of rs4612666 C/T was negatively associated with the presence of ANA in primary SS. On the contrary, anti-Ro antibodies were detected without statistical significance in relation to the genotypes or allele frequency of the NLRP3 gene.

## 4. Discussion

NLRP3 senses foreign pathogens and various stimuli to form and activate the NLRP3 inflammasome. The central function of the NLRP3 inflammasome is to activate caspase-1 and pro-inflammatory cytokines, especially IL-1β and IL-18 [2,3]. NLRP3 inflammasome activity and downstream inflammasome-related protein molecules are upregulated in RA and SS patients [8,18]. The genetic polymorphisms of NLRP3 have been observed to serve a vital role in the disease susceptibility and severity of different autoimmune diseases [13,14,26]. However, the biological role of the NLRP3 gene in the immunopathogenesis of autoimmune diseases has been little documented. We have investigated several polymorphic sites on the NLRP3 gene in RA and primary SS patients. The results indicated that the T allele of rs4612666 C/T elevated the risk of RA. Intriguingly, Cheng et al. demonstrated that the C allele of rs4612666 and the G allele of rs10754558 were associated with a significantly increased risk of RA in Han Chinese [20]. One of the possibilities of the discrepancy in results might be due to the collected sample population, or the Taiwanese population is probably not a single ethnicity. The T allele was shown to serve as a risk factor to alter the susceptibility to RA disease. Notably, we found that the C allele of genotype TC rs12137901 was also more susceptible to primary SS in HLA-DR8-negative patients. This result suggested that the C allele of genotype TC rs12137901 is a potential risk factor for pSS HLA-DR8 negative individuals.

In the clinical features of the tested cases, we noticed that NLRP3 genotypes potentially influenced the clinical outcome and progression of diseases. Although genotype rs12079994 G/A was not related to the susceptibility of RA, the A allele was positively associated with the high titer of RF and high concentration of autoantibodies in RA and primary SS patients, respectively. The RF titer was significantly high in RA patients with A allele of rs12079994 G/A polymorphism. We also found that the G allele of genotype rs10754558 G/G was in a high amount of anti-CCP antibody in RA patients. Antinuclear antibody and anti-CCP antibody were observed to have a positive association with A allele of rs12079994 G/A polymorphism in primary SS. These results suggested that the potential allele effect accelerated the progression of disease. On the other hand, the C allele of rs4612666 C/T was negatively associated with the presence of ANA in primary SS. C allele negative of genotype rs4612666 C/T was also in the high odds ratio of anti-CCP antibody, although without significance. On the contrary, the C allele of rs4612666 C/T contributes an ameliorative effect of autoantibodies production.

The production of cytokines and autoantibodies is characterized in the pathogenesis of autoimmune diseases [1,17]. We observed that RA patients exhibited high titer of RF antibody and anti-CCP antibody, whereas primary SS patients expressed anti-CCP and ANA antibodies. Sjogren syndrome is characterized by lymphocyte and epithelial inflammation and dysfunction of secretory glands. Primary SS patients generally expressed antinuclear antibodies and anti-Ro antibodies; however, anti-Ro antibodies were not detected with significance in relation to the genotypes or allele frequency of the NLRP3 gene in this study (data not shown). Previous studies reported that autoantibodies activated the activity of NLRP3 inflammasome and exacerbated the severity of autoimmune diseases [1,29]. Experimental animal model evidence also demonstrated that injection of anti-dsDNA antibodies from systemic lupus erythematosus patients promoted the disease progression in mice, and the Th17/Treg cell ratio was also increased [30,31]. Anti-dsDNA reversely activated NLRP3 inflammasome by binding to toll-like receptor 4 in monocytes [31]. Serum thyroid peroxidase antibody and thyroglobulin antibody are detected in association with the activation of NLRP3 in autoimmune thyroiditis patients [32]. Taking this evidence together, NLRP3 polymorphisms are related to autoimmune diseases and the activation of inflammasome [1]. Moreover, the integrative analysis of gene polymorphisms and disease susceptibility using different systems approaches or single-cell multi-omics data might further uncover biological and regulatory insights into risk genes [33,34]. In conclusion, we have shown that NLRP3 polymorphisms play a critical role in the pathogenesis of autoimmune diseases. The disease severity of RA can be partially explained by the activity of NLRP3 inflammasome and NLRP3 gene polymorphisms. Recently, the therapeutics development of autoimmune diseases has been designated to target NLRP3 inflammasome. There is evidence suggesting that inhibition of the formation and activation of NLRP3 is an effective therapeutic strategy. This study provides additional information on NLRP3 gene polymorphisms’ association with disease status, and certainly, further experiments are still required.

## 5. Conclusions

The activation of NLRP3 inflammasome leads to the stimulation of cytokines and is significantly involved in the pathogenesis and progression of autoimmune diseases. We noticed that NLRP3 genotypes potentially influenced the clinical outcome and progression of RA and pSS. NLRP3 polymorphisms play a vital role in the pathogenesis of autoimmune diseases, and that disease severity can be partially explained by the activity of NLRP3 inflammasome. This study provides additional information on the relationship between NLRP3 gene polymorphisms and autoimmune disease and may be evidence for further experiments investigating the role of NLRP3 inflammasome in autoimmune diseases.

## Figures and Tables

**Table 1:** Genotypes and allele frequency of NLRP3 gene in RA patients.

			RA Cases (*n* = 239)	Control Cases (*n* = 170)	*p*	OR (95% CI)
**rs12079994**	**Genotype**	GG	178	121		1
		GA	54	47		0.78 (0.50–1.23)
		AA	7	2		2.38 (0.49–11.65)
	**Dominant**	GG	178	121		1
		GA + AA	61	49		0.85 (0.54–1.32)
	**Recessive**	GG + GA	232	168		1
		AA	7	2		2.53 (0.52–12.35)
	**Allele Frequency**	G	410	289		1
		A	68	51		0.94 (0.63–1.39)
**rs12137901**	**Genotype**	TT	108	89		1
		TC	104	70		1.22 (0.81–1.85)
		CC	27	11		2.02 (0.95–4.30)
	**Dominant**	TT	108	89		1
		TC + CC	131	81		1.33 (0.90–1.98)
	**Recessive**	TT + TC	212	159		1
		CC	27	11		1.84 (0.89–3.82)
	**Allele Frequency**	T	320	248		1
		C	158	92		1.33 (0.98–1.81)
**rs4612666**	**Genotype**	CC	56	56		1
		CT	125	84		1.49 (0.94–2.36)
		TT	58	30	0.024	1.93 (1.09–3.44)
	**Dominant**	CC	56	56		1
		CT + TT	183	114	0.034	1.61 (1.04–2.49)
	**Recessive**	CC + CT	181	140		1
		TT	58	30		1.50 (0.91–2.45)
	**Allele Frequency**	C	237	196		1
		T	241	144	0.023	1.38 (1.05–1.83)
**rs10754558**	**Genotype**	CC	97	58		1
		CG	107	84		0.76 (0.49–1.17)
		GG	35	28		0.75 (0.41–1.35)
	**Dominant**	CC	97	58		1
		CG + GG	142	112		0.76 (0.50–1.14)
	**Recessive**	CC + CG	204	142		1
		GG	35	28		0.87 (0.51–1.49)
	**Allele Frequency**	C	301	200		1
		G	177	140		0.84 (0.63–1.12)

OR: odds ratio. CI: confidence interval.

**Table 2:** Genotypes and allele frequency of NLRP3 gene in primary SS patients.

			Primary SS Cases (*n* = 285)	Control Cases (*n* = 170)	*p*	OR (95% CI)
**rs12079994**	**Genotype**	GG	216	121		1
		GA	68	47		0.81 (0.53–1.25)
		AA	1	2		0.28 (0.03–3.12)
	**Dominant**	GG	216	121		1
		GA + AA	69	49		0.79 (0.51–1.21)
	**Recessive**	GG + GA	284	168		1
		AA	1	2		0.30 (0.03–3.29)
	**Allele Frequency**	G	500	289		1
		A	70	51		0.79 (0.54–1.17)
**rs12137901**	**Genotype**	TT	129	89		1
		TC	134	70		1.32 (0.89–1.96)
		CC	22	11		1.38 (0.64–2.99)
	**Dominant**	TT	129	89		1
		TC + CC	156	81		1.33 (0.91–1.94)
	**Recessive**	TT + TC	263	159		1
		CC	22	11		1.21 (0.57–2.56)
	**Allele Frequency**	T	392	248		1
		C	178	92		1.22 (0.91–1.65)
**rs4612666**	**Genotype**	CC	56	56		1
		CT	125	84		0.86 (0.56–1.33)
		TT	58	30		1.14 (0.66–1.98)
	**Dominant**	CC	56	56		1
		CT + TT	183	114		0.94 (0.63–1.40)
	**Recessive**	CC + CT	181	140		1
		TT	58	30		1.24 (0.77–2.02)
	**Allele Frequency**	C	237	196		1
		T	241	144		1.04 (0.79–1.37)
**rs10754558**	**Genotype**	CC	99	58		1
		CG	138	84		0.96 (0.63–1.47)
		GG	48	28		1.00 (0.57–1.77)
	**Dominant**	CC	99	58		1
		CG + GG	186	112		0.97 (0.65–1.45)
	**Recessive**	CC + CG	237	142		1
		GG	48	28		1.03 (0.62–1.71)
	**Allele Frequency**	C	336	200		1
		G	234	140		0.99 (0.76–1.31)

OR: odds ratio. CI: confidence interval.

**Table 3:** Genotypes and allele frequency of NLRP3 gene in HLA-DR8 pSS patients.

			HLA-DR8 (+)		*p*	OR (95%CI)	HLA-DR8 (−)		*p*	**OR (95%CI)**
			Sjogren’s Syndrome Cases	Control Cases			Sjogren’s Syndrome Cases	Control Cases		
			(*n* = 68)	(*n* = 15)			(*n* = 120)	(*n* = 97)		
**rs12137901**	**Genotype**	TT	41	8		1	47	52		1
		TC	24	6		0.78 (0.24–2.52)	61	40		1.69 (0.96–2.96)
		CC	3	1		0.59 (0.05–6.37)	12	5		2.66 (0.87–8.10)
	**Dominant**	TT	41	8		1	47	52		1
		TC + CC	27	7		0.75 (0.24–2.32)	73	45	0.034	1.79 (1.04–3.09)
	**Recessive**	TT + TC	65	14		1	108	92		1
		CC	3	1		0.65 (0.06–6.68)	12	5		2.04 (0.69–6.02)
	**Allele Frequency**	T	106	22		1	155	144		1
		C	30	8		0.78 (0.31–1.92)	85	50	0.031	1.58 (1.04–2.40)

OR: odds ratio. CI: confidence interval.

**Table 4:** Associations of alleles of NLRP3 genotype with anti-CCP antibody in primary Sjogren’s syndrome patients.

		Anti CCP (+) (*n* = 12)	Anti CCP (−) (*n* = 173)	*p*	OR (95% CI)
**rs12079994**	A Allele Positive	6	33	0.011	4.24 (1.29–13.99)
	A Allele Negative	6	140		
**rs4612666**	C Allele Positive	7	142	**NS**	
	C Allele Negative	5	31		**3.27 (0.97–10.99)**

**Table 5:** Associations of alleles of NLRP3 genotype with ANA in primary Sjogren’s syndrome patients.

		ANA (+) (*n* = 92)	ANA (−) (*n* = 109)	*p*	OR (95% CI)
**rs12079994**	A Allele Positive	25	17	0.044	2.02 (1.01–4.03)
	A Allele Negative	67	92		
**rs4612666**	C Allele Positive	67	94	0.018	
	C Allele Negative	25	15		2.34 (1.15–4.77)

ANA: anti-nuclear antibody.

## Data Availability

No new data were created or analyzed in this study. Data sharing is not applicable to this article.
